# Fibrin, from blood to bone: a review

**DOI:** 10.1016/j.bonr.2026.101925

**Published:** 2026-05-30

**Authors:** Hamza Danguir, Lisa Reiniche, Khalil El Mabrouk, Maxime Ducret, Meriame Bricha, Mourad Bekhouche

**Affiliations:** a Laboratory of Tissue Biology and Therapeutic Engineering (LBTI), UMR CNRS 5305 Université Lyon 1, 69007, Lyon, France; b Euromed University of Fes, UEMF, 30030, Fes-Meknes, Morocco

**Keywords:** Fibrin, Bone, Mechanobiology, Tissue repair, Biomaterial, Good Health and Well-being

## Abstract

Fibrin is principally involved in blood coagulation and hemostasis. Its structure, abundance and turnover are reciprocally related to blood flow. Decreased abundance of fibrin in afibrinogenemic or hemophilic patients leads to bone disorders such as bone cysts or hemarthroses. Fibrin accumulation in growing children with chronic disease leads to osteoarthritis and physeal disruption. Despite its clear role in bone disorders; the underlying mechanisms are not clearly understood. Bone formation and hemostasis is mediated by osteocyte mechanosensitivity involving plasma membrane disruption, calcium signaling cascade. Fibrin is related to mechanotransduction in platelets and recently in mitochondrial mechanosensing in macrophages, however its role in bone mechanobiology is not elucidated. This review underlines the link of fibrin and bone pathophysiology and emphasizes the gap of knowledge between fibrin function during healing and mechanobiology of bone homeostasis. This review proposed an original perspective to better understand bone loss, repair and formation, that could open the path to further investigations to adapt innovative therapeutic strategies.

## Highlights

• Fibrin plays pathophysiological roles in bone homeostasis and osteoarticular disorders.
• Fibrin functions in blood hemostasis and tissue repair are related to mechanobiological.
• The role of fibrin in bone mechanobiology remains to be elucidated.
• Fibrin can be used as a 3D scaffold for bone tissue repair.

## 1 Introduction

Whenever an injury occurs, the body naturally regenerates damaged tissue by mobilizing a large panel of proteins and enzymes (Kenkre and Bassett, 2018; Hadjidakis and Androulakis, 2006). This restoration involves interactions among cells, the extracellular matrix (ECM), and signaling molecules (Gurtner et al., 2008). Among these wound healing actors, fibrin plays an early and major role at the injury site. It starts as inactive fibrinogen secreted into the bloodstream and is later activated at the healing site, forming a 3D ECM (Mosesson et al., 2001). Several reviews report precisely the mechanisms of wound healing notably by focusing on skin tissue (Mosesson et al., 2001; Weisel and Litvinov, 2017; Collen and Lijnen, 1991; Thompson et al., 1985; McKenzie et al., 1975; Medcalf, 2007; Levi et al., 2004; Liu et al., 2006). This review focuses on the role of fibrin at the initial steps of tissue repair and on the formation of osteoarticular tissue which are various forms related to blood and fibrin supply in pathophysiological situations.

Fibrin is involved in tissue repair going from soft (skin) to hard (bone) connective tissues (Ducret et al., 2021). Bone formation is a paradigm for illustrating the role of fibrin and blood supply in the formation of connective tissue of various forms (Marenzana and Arnett, 2013; Lee et al., 2014). In the osteochondral block the connective tissue structure is correlated with blood supply and oxygen with hypoxic conditions found in cartilage (Oliveira et al., 2021). Microfracture is a surgical technique based on blood supply for cartilage repair supporting the assumption that fibrin constitutes a biomolecule naturally adapted to promote osteoarticular tissues repair. Fibrin structure and abundance is related to bone disorders such as bone cysts, hemarthrose, osteoarthritis and physeal disruption (de Moerloose et al., 2013; Charbit et al., 2007; Cole et al., 2014, Cole et al., 2021; Vilar et al., 2020). These functions are closely related to blood flow. Blood clot structure and concentration are regulated *in vivo* by the hydrodynamic shear from physiological flow of blood resulting in a higher protein content at higher flow (Risser et al., 2022). Intravascular thrombus is also controlled by the physiological shear promoting resistance to fibrinolytic degradation (Whyte and Mutch, 2022). How fibrin influences bone homeostasis is not fully understood.

Bone is a dynamic tissue that constantly adapts to its physical environment (Yavropoulou and Yovos, 2016). When the skeleton is subject to specific loads and physical stresses, the bone adapts through a site-specific response. Against major stress, the bone becomes stronger and larger (Yavropoulou and Yovos, 2016; Karlsson et al., 1993). In contrast, low stress makes the bone weaker (Yavropoulou and Yovos, 2016). Cells convert mechanical and physical energy into biochemical signals through mechanosensitive signaling pathways (Yavropoulou and Yovos, 2016). Osteocytes play a central role in the efficient regulation of bone remodeling cycle. They are the most abundant cells in bone, embedded within the lacunocanalicular network able to sense interstitial fluid flow due to mechanical loading (Yavropoulou and Yovos, 2016). These mechanical stimulations are required for osteocytes viability (Bonewald, 2006). Furthermore, strong blood supply is required to control bone adaptation mechanisms, to spatially and temporally guide these adaptations (Yavropoulou and Yovos, 2016). Blood flow delivers nutrients, oxygen and signaling molecules to osteocytes, to sustain their function, and efficiently regulate the bone remodeling cycle (Yavropoulou and Yovos, 2016). Mechanical stimulation such as physiological loading induces increased fluid flow, plasma membrane disruptions, subsequent calcium signaling, bone formation and reduced sensitivity to loading by the expression of protecting proteins such as sclerostin (Yu et al., 2025; Verbruggen et al., 2014a; Rolvien and Amling, 2022; Jing et al., 2014; Hagan et al., 2021, Hagan et al., 2024). Conversely, bone disuse such as bed rest or space flight leads to bone loss or even osteoporosis (Yu et al., 2025; Rolvien and Amling, 2022).

In addition to being a biomolecule whose function is related to blood flow, fibrin is reported to be related to mechanotransduction in platelets, and recently in macrophages in a 3D fibrin biomaterial (Goncalves et al., 2005; Gao et al., 2025). Despite these data, the role in bone formation and homeostasis, at a molecular and cellular level, is not elucidated. Fibrin is used as a scaffold for regenerative medicine and tissue engineering (Ducret et al., 2021). The fibrin matrix exhibits excellent biocompatibility and can be tuned to carry other bioactive molecules, such as cells, drugs, and other biomaterials, to deliver them directly to the healing site in a controlled manner, as a hydrogel (Ducret et al., 2021; Noori et al., 2017). In this context, fibrin was first introduced in 1970 as a sealant for surgical applications (Matras, 1985). Its employment was basic, used as a glue to promote hemostasis and tissue adhesion. Afterwards, fibrin hydrogel emerged as a biomaterial promoting tissue repair and was proposed as a tunable carrier of mineralizing material for mineralized tissue regeneration for its ease of administration as an injectable, with a fine needle, to reach deep tissues (Ducret et al., 2021; Rojas-Murillo et al., 2022). However, its role in mechanobiological formation and homeostasis of bone might be better understood to promote tissue repair and avoid bone toxicity.

This review reports the roles of fibrin in bone regeneration, providing an original perspective as a biomolecule provided from blood with a spatiotemporally controlled abundance during the wound healing process. The first part highlights the biological properties of fibrin and its roles in the human body, with the associated cells. The second part reports the mechanobiology of bone where abundance and structure of fibrin is related to the pathophysiological state including the example of the osteochondral block. The last part highlights the possible ways to use fibrin-based biomaterials for osteoarticular regeneration.

## 2 Results and discussion

### 2.1 Fibrin synthesis

**Fig. 1:**
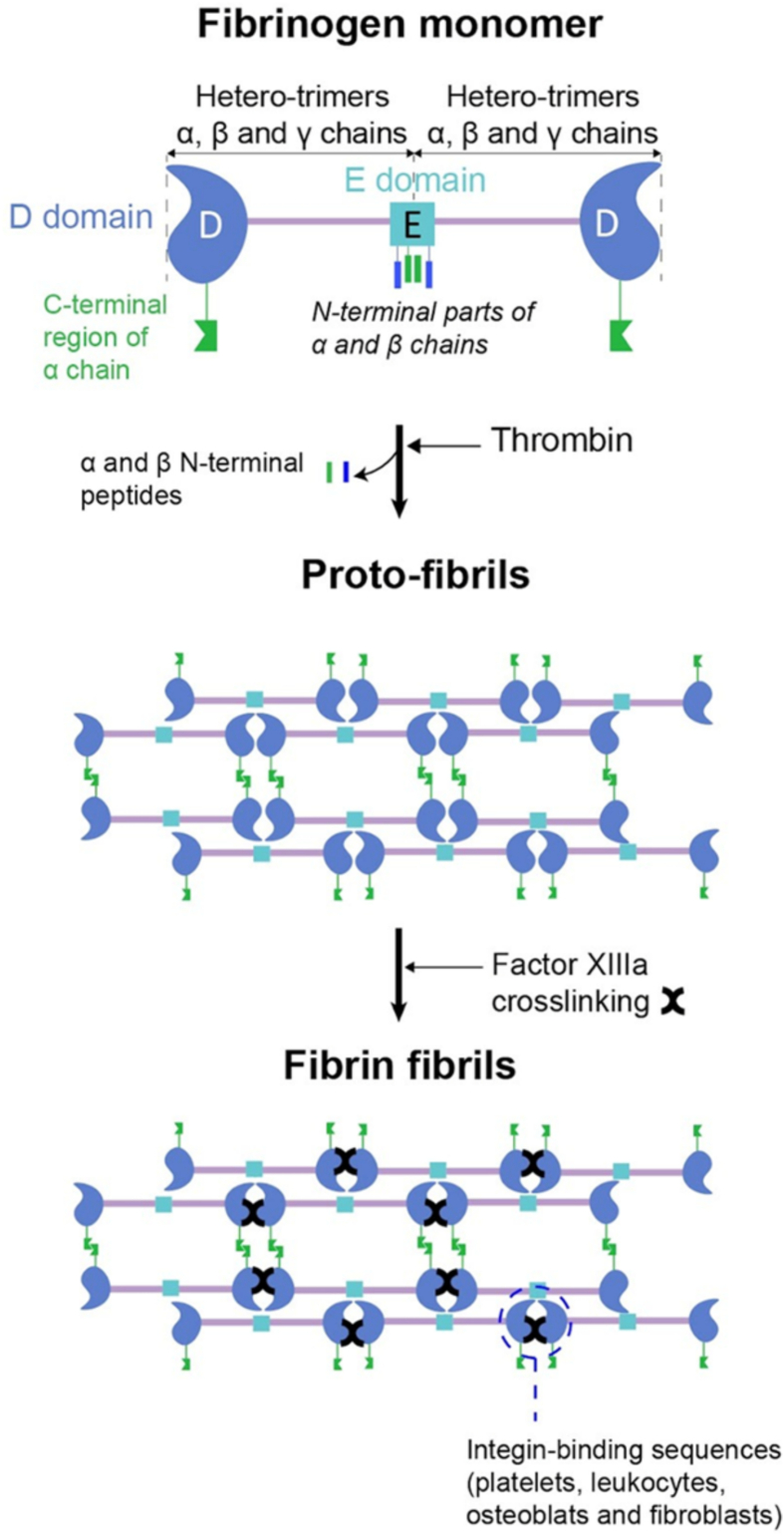
Fibrin structure and domain organization.

Fibrin is a high molecular weight (340 kDa) glycoprotein composed of three chains named alpha (Fibrinogen alpha chain, α), beta (Fibrinogen beta chain, β), and gamma (fibrinogen gamma chain, γ) chains encoded by three clustered genes, located in the human chromosome 4 (FGA, FGB, and FGG) (Wolberg, 2023). Fibrin trimers form a quaternary structure based on the association of their N-terminal part, forming a common E-domain in the hexamer form (dimer of trimer). Two globular C-terminal D-domains are located at the extremity of the beta and gamma chains. The C-terminal part of the alpha chain forms a tail emanating from the D-domains and ending with a small globular αC-domain (Fig. 1).

Fibrin is synthesized in hepatocytes in the form of soluble fibrinogen and secreted into the bloodstream at high concentrations (2–4 mg/mL) (Vilar et al., 2020; Wolberg, 2023; Woodson et al., 2013). Fibrin exposure of the connective tissue induces the recruitment of platelets and the activation of a series of proteolytic events, including serine proteases and the so-called coagulation cascade (Swieringa et al., 2018; Farges et al., 2015). Fibrinogen undergoes a proteolytic maturation by the removal of the N-terminal fibrinopeptides from the α (FpA) and the β (FpB) chains by a specific serine protease, thrombin (Weisel and Litvinov, 2017) (Fig. 1). FpA is cleaved at the Arg16-Gly17 position, while FpB is cleaved at the Arg14-Gly15 position (Vilar et al., 2020). This process involves longitudinal interactions between the D-domains of two fibrin hexamers, forming D—D dimers, as well as lateral associations between these D—D dimers and knob A structures in the E-domain, which are exposed following fibrinopeptide cleavage (Weisel and Litvinov, 2017). The final fibrin polymer is then stabilized by the transglutaminase factor XIII, a protein that is also activated by thrombin to become factor XIIIa (Periayah et al., 2017). Factor XIIIa links fibrin monomers to each other by catalyzing the formation of covalent bonds between fibrin chains, notably the D-dimers (Wolberg, 2023). Fibrin is involved in the later stage of hemostasis. It forms a temporary ECM to fulfill different host tissues (Woodson et al., 2013). The proteolytic mechanisms derived from platelets, the endothelium, and the injured tissue (specific or stem/progenitor cells) are implicated in fibrin formation to trap blood cells. Proteolytic activities are also crucial for fibrin degradation and its replacement by repaired tissue (Collen and Lijnen, 1991). This ECM provides amino acid motifs or peptides, either sequestered or released, that allow cell activation, such as the Arg-Gly-Asp (RGD) sequence needed for integrin-mediated signaling (Mosesson, 2005). These cleavage events result in the release of fibrin degradation products, including D-dimer, the E-domain, and the αC-domain, which are related to angiogenesis (Thompson et al., 1985) and chemotaxis (McKenzie et al., 1975), and therefore to the inflammatory response (Medcalf, 2007; Levi et al., 2004). Originating from blood, fibrin harbors signals and biomechanical properties to allow hemostasis and bone repair.

### 2.2 Fibrin biological function and associated cells

**Fig. 2:**
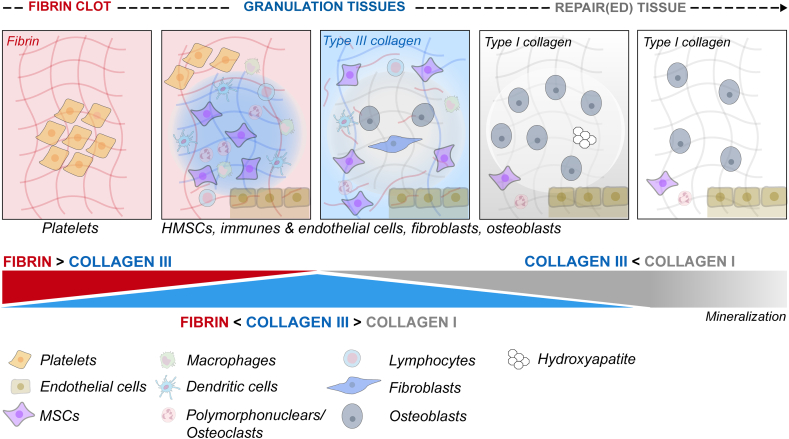
Schematic representation of ECM replacement during tissue healing. Healing starts with the formation of a fibrin-rich temporary matrix, followed by a collagen type III-rich matrix with fibrin degradation corresponding to granulation tissue. This matrix is subsequently replaced by a collagen type I-rich matrix, ultimately leading to a mature mineralized matrix containing osteocytes.

Fibrin is involved in many biological mechanisms, including tissue repair, regeneration, and blood clotting (Fig. 2). This protein has variable biomechanical properties, including extensibility and elasticity, viscoelastic behavior, and stiffness, which induce tunable, reciprocal stress behavior according to cell strain (Weisel and Litvinov, 2017; Liu et al., 2006).

Fibrin plays a crucial role in tissue healing. This process consists of 4 stages. Homeostasis occurs in the first step, which involves forming an immediate fibrin-based clot and vasoconstriction to prevent blood loss (Humphrey and Schwartz, 2021; Laurens et al., 2006). In the blood clot, fibrin serves as a matrix for cell binding and migration. Several cells bind fibrin through integrin receptors such as integrin αIIbβ3 on platelets (Höök et al., 2017), integrin αMβ2/Mac-1 on leukocytes (mast cells, monocytes, macrophages, and neutrophils) (Gailit et al., 1997), or integrin αVβ3, present on osteoblasts, fibroblasts, and endothelial cells (Flick et al., 2004; Gawaz et al., 1997). Cell binding to fibrin triggers signaling pathways, such as focal adhesion kinase (FAK) and actin polymerization for cell migration (Martin et al., 2002; Zhao and Guan, 2011), and RAS/MAPK signaling for ECM remodeling involving metalloproteases (Eliceiri and Cheresh, 1999). After hemostasis, inflammation occurs, involving proteolytic cascades, including matrix metalloproteinases, and the protease plasmin, which plays a central role in fibrin degradation.

The fibrinolytic cascade is initiated by plasmin (Collen and Lijnen, 1991), which is mainly expressed by endothelial cells (Dhahri et al., 2016; Deryugina and Quigley, 2012). Plasmin activity is also at the center of multiple proteolytic events that foster tissue remodeling, such as the activation of pro-matrix metalloproteinases (proMMPs) into active MMPs (MMP-1, MMP-2, MMP-3, MMP-7, MMP-8, MMP-9, MMP-12, MMP-13, MMP-14) (Hiller et al., 2000; Lelongt et al., 2001). Plasmin and MMPs release several latent cytokines, chemokines, and growth factors (Manicone and McGuire, 2008). The fibrin-associated plasmin protease also activates the uPA/uPAR signaling in platelets, monocytes, macrophages, fibroblasts, endothelial cells, and bone marrow-derived mesenchymal stem cells (BD-MSCs) (Heissig et al., 2015; Neuss et al., 2009). This activation promotes matrix remodeling, notably bone tissue repair, monocyte invasion, and their differentiation into macrophages, through multiple signaling pathways, including RAS-ERK1/2, p38 MAPKK, and RhoA-ROCK (Deryugina and Quigley, 2012; Heissig et al., 2015). Released inflammatory mediators and cytokines, from thrombocytes and other inflammatory cells (neutrophils, monocytes, macrophages, and mast cells) participate in removing damaged cells and bacteria from the site for cleaning and tissue neosynthesis (Wallace et al., 2025).

Tissue degradation and synthesis require cell proliferation and migration; this is the granulation stage, which overlaps with the inflammatory stage. The clot is replaced by secondary, temporary tissue called granulation tissue, rich in type III collagen, a homotrimeric, simple fibrous collagen that is thinner and more sensitive to proteolysis than type I collagen (Singh et al., 2023). Fibrin plays a critical role in supporting the formation of this temporary, high-cell-density granulation tissue (Laurens et al., 2006), which contains tissue-specific cells (osteoblasts, keratinocytes, fibroblasts…), endothelial cells, and immune cells. Furthermore, MSCs are present in the system, accounting for 0,001-0,01% of bone marrow mononuclear cells (Bhat et al., 2021), and participate in general tissue homeostasis and repair under the action of chemokine factors such as CXCL12, which is the dominant chemokine that controls the stem cells pathways in bone marrow (Antoniou et al., 2010). Granulation tissue is highly vascularized to provide nutrients and energy to various cells and to ensure sufficient drainage for tissue replacement and, notably, to clear the environment. The final stage of wound healing is the remodeling phase. This phase is characterized by the degradation of immature and excess collagen, specifically type III collagen, that is left disorganized and weak (Alhajj and Goyal, 2026; Mathew-Steiner et al., 2021). This degradation is caused by proteolytic enzymes, mainly MMPs (Mathew-Steiner et al., 2021). Wound contraction also occurs at this stage to a much greater extent, driven by fibroblasts that transition to myofibroblasts, which reorganize the new ECM (Tomasek et al., 2002). Vascular endothelial growth factor (VEGF) is highly abundant at this phase and contributes to ECM remodeling by promoting angiogenesis, thereby ensuring nutrient and oxygen supply to the wound. Finally, type I collagen replaces type III collagen (Singh et al., 2023).

In bone repair, type I collagen is deposited by osteoblasts and mineralized to form bone mineralized tissues. This mechanism is controlled by inflammatory mediators and receptors and their ligands, osteoprotegerin (OPG) and RANK, to shift the equilibrium toward bone apposition (Udagawa et al., 2021). During bone damage, fracture healing inevitably recruits fibrin into the site (Yuasa et al., 2015), initiating the bone regeneration. This temporary matrix facilitates the recruitment and adhesion of osteoprogenitor cells, creating an osteogenic environment conducive to bone restoration. However, continuous fibrin recruitment in the fracture site could be related to pathologic bone disease, altering normal hemostasis (de Moerloose et al., 2013; Bugge et al., 1996). Abnormal fibrin accumulation leads to an increase in the local inflammation, which results in pathological bone alteration, such as osteoporosis (Cole et al., 2014). Blood flow plays a critical role in fibrin biological functions notably by controlling integrin αIIbβ3 mechanotransduction for shear activation of platelets, as demonstrated by Goncalves et al. (2005). Furthermore, this study showed that this force-dependent activation also requires cytosolic Ca^2+^ influx, triggered by sudden acceleration in blood flow, which makes blood flow critically important in this context (Goncalves et al., 2005). Increasing the hydrodynamic shear from physiological flow of blood leads to a higher fibrin protein content within the blood clot (Risser et al., 2022). The physiological shear strengthens intravascular thrombus by promoting resistance to fibrinolytic degradation (Whyte and Mutch, 2022). Taken together, these data suggest that the distinction between physiological fibrin deposition and pathological fibrin accumulation is critical when interpreting fibrin's role in the human skeleton.

There are different types of drugs known to have a harmful impact on bone, and assessing whether fibrin abundance in these contexts reflects adaptive repair or adverse skeletal remodeling requires a translational framework that integrates multiple assessment modalities (Tuladhar et al., 2025). For instance, abnormal glucocorticoid exposure influences trabecular bone microarchitecture, with major decrease in serum biomarkers of both bone formation and resorption, ultimately leading to skeletal deterioration and bone toxicity (Tuladhar et al., 2026). In these patterns of disrupted remodeling, fibrin turnover may play a contributing role, highlighting the fact that fibrin should not be interpreted solely as a repair protein, since its abundance is highly context-dependent. Table 1 summarizes the mechanotransduction linked to fibrin through fluid flow, and linked to bone, through osteocytes pathways (Table 1).

**Table 1:** Fibrin and osteocytes in mechanotransduction and bone repair concepts.

Mechanotransduction pathways	Fibrin kinetics	Osteocyte
Fluid flow	Fibrinolysis is modulated by fluid shear stress: flow accelerates plasmin-driven fibrin degradation Stasis promotes fibrin persistence (Whyte and Mutch, 2022)	Mechanical loads triggers interstitial fluid flow through lacuno-canalicular network Mechanical loads are the primary signal for osteocytes (Verbruggen et al., 2014b; Muñoz et al., 2025; You et al., 2008)
	Stimulation of fluid flow determines structural and mechanical properties of fibrin (Risser et al., 2022)	Reduced osteocyte connectivity disrupts fluid flow, and impairs Ca^2+^ mechanosensing (Yu et al., 2025; Schurman et al., 2021)
	Reduced flow impairs fibrinolysis, and causes fibrin accumulation (Whyte and Mutch, 2022)	Disuse reduces fluid flow and reduces osteocyte signaling, which favours bone resorption (Rolvien and Amling, 2022; Jing et al., 2014; Hughes et al., 2023)
Plasma membrane disruptions	NINJ1-dependant PMD triggers fibrin coagulation and inflammation (Cui et al., n.d.)	Fluid shear stress triggers PMDs in osteocytes, repaired by Prkd1 Unrepaired PMDs impairs bone remodeling (Hagan et al., 2021; Tuladhar et al., 2024)
Bone toxicity	Intra-articular abnormal fibrin causes bone and cartilage toxicity Fibrin persistence impairs repair kinetics (Charbit et al., 2007)	Drug-induced bone toxicity disrupts osteocytes survivability and lacuno-canalicular network, impairing bone fluid flow (Tuladhar et al., 2025)
	Glucocorticoids impairs fibrinolysis by altering PAI-1/tPA balance (Risser et al., 2022)	Glucocorticoids are responsible for osteocyte apoptosis, and lacuno-canalicular network disruption (Tuladhar et al., 2025) Glucocorticoids impairs bone fluid flow (Rolvien and Amling, 2022; Tuladhar et al., 2024)

### 2.3 Bone formation

**Fig. 3:**
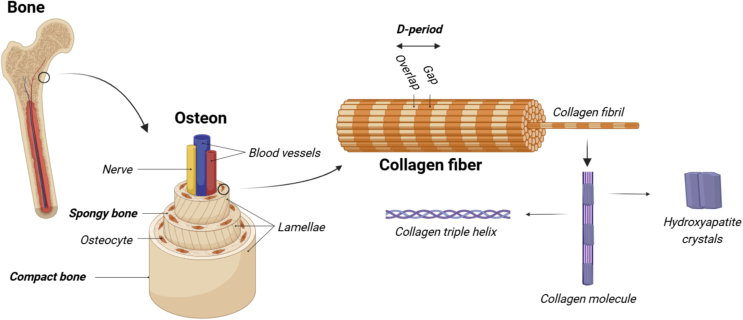
Schematic of the hierarchical structure of bone from the whole bone to nanostructure. Bone is a vascularized tissue, composed of compact and spongy bone, and containing osteons (Harvesian systems). At the tissue level, bone is organized into lamellae, surrounding osteonic canals. At the microscale, collagen fibers are organized in collagen fibrils. At the nanoscale, hydroxyapatite crystals are embedded in collagen triple helix fibrils, creating a natural composite.

Natural bone tissue is comparable to a composite material, consisting of an organo-mineral matrix and bone cells. The average composition of natural bone is 70% mineral phase, 10% water, and 20% organic matter. The mineral phase is composed of poorly crystallized carbonate hydroxyapatite, while the organic phase is made up of 95% collagen, a fibrous glycoprotein that acts as the bone matrix (Fig. 3) (Brochu et al., 2024). Bone cells are composed of two components: osteoblasts, which build bone mass, and osteoclasts, which break it down. The activity of these two cell types is in perpetual synergistic action to maintain the skeletal structure and thus ensure bone remodeling. Bone tissue can be divided into two categories: i- Cortical or compact bone: a hard, solid tissue formed of osteons interspersed with bone lamellae. ii- Spongy bone: less hard, it is formed of bone lamellae that delineate small cavities.

Bone is an isolated tissue, surrounded by a layer of osteoblasts connected by tight and gap junctions, and is continuously renewed and remodeled through the bone remodeling cycle. Osteoblasts, osteoclasts, and osteocytes are all actors in this cycle, through coordinated actions (Florencio-Silva et al., 2015). Osteoblasts originate from MSCs, which require specific genes to commit to the osteoprogenitor lineage (Grigoriadis et al., 1988). Genes such as osterix (Osx), distal-less homeobox 5 (Dlx5), and Runt-related transcription factors 2 (RUNX2) are necessary for osteoblast differentiation (Florencio-Silva et al., 2015). RUNX2 upregulates other genes related to osteoblasts, such as Alkaline Phosphatase (ALP), osteocalcin (OCN), and collagen type 1 alpha 1 (Col1A1) (Fakhry et al., 2013). Osteoblasts synthesize bone tissue by first producing collagen proteins, proteoglycans (decorin and biglycan), and noncollagen proteins (OCN, osteonectin, BSP II, and osteopontin (OPN)). These components form the organic matrix of bone, which is then mineralized by calcium ions stored within matrix vesicles, forming hydroxyapatite crystals in association with phosphate released by ALP activity. Hydroxyapatite then spreads into the matrix (Florencio-Silva et al., 2015). The mineralization process is controlled by small integrin-binding ligand N-linked glycoproteins (SIBLINGs), which include three sialoproteins (dentin sialophosphoprotein DSPP, matrix extracellular phosphoglycoprotein MEPE, dentin matrix protein 1 DMP1, and bone sialoprotein BSP), which directly interact with calcium ions and calcium phosphate (Staines et al., 2012). The SIBLINGs are also involved in the formation of other mineralized tissues such as dentin and enamel. Osteoblasts can be included in the mineralized space, forming osteocytes with a dendritic morphology. Osteocytes form an interconnected network, promoting cell-cell communication and acting as mechanosensors to help the bone adapt to daily mechanical forces, through mechanotransduction (Rochefort et al., 2010). These cells translate shear stress into biochemical signals, critically needed for bone homeostasis (Yu et al., 2025). In this context, stresses cause plasma membrane disruptions (PMDs), which initiate mechanotransduction and calcium signaling (Jing et al., 2014; Hagan et al., 2024; Tuladhar et al., 2024). PMDs correlation to fibrin and bone are reported in Table 1, highlighting their relevance (Table 1). Calcium acts as an intracellular signaling molecule, released whenever osteocytes are mechanically stimulated, and propagated across the osteocyte network through gap junctions (Udagawa et al., 2021), confirming that bone remodeling is as much a cell signaling event as it is a matrix event. Mechanosensitivity depends on the fragility and vulnerability of the osteocyte's plasma membrane, regulated by the expression of extracellular protein such as sclerostin or by cytoskeletal proteins such as β2-spectrin (Rolvien and Amling, 2022; Hagan et al., 2021, Hagan et al., 2024). A deficiency in this specific protein disrupts the balance, increasing the number of PMDs, which impairs cell survival. Furthermore, osteocytes regulate the bone remodeling cycle by controlling both osteoblastic and osteoclastic activities (Dallas et al., 2013). Several genes implicated in bone synthesis, such as OCN, ALP, and collagen type I, are downregulated in osteocytes, while genes implicated in bone regulation, such as DMP1 and sclerostin, are upregulated (Florencio-Silva et al., 2015).

Osteoclasts are derived from hematopoietic stem cells (HSCs), under the influence of several factors, including macrophage colony-stimulating factor (M-CSF) secreted by osteoblasts and osteoprogenitor mesenchymal cells, and RANKL, synthesized by osteoblasts and stromal cells (Florencio-Silva et al., 2015; Boyce et al., 1999). When RANKL is bound to its receptor RANK, located on osteoclastic precursor cells, and when M-CSF is bound to its receptor, colony-stimulating factor-1 receptor (C-FMS), osteoclast differentiation is triggered. Osteoprotegrin (OPG), an inactive soluble receptor, is a factor capable of inhibiting osteoclastogenesis by antagonising RANK and preventing the RANK/RANKL interaction (Udagawa et al., 2021). Osteoclasts attach to the bone tissue surface under the influence of αvβ3-integrin and CD44 (Florencio-Silva et al., 2015). This ruffled border contains a vacuolar-type H + -ATPase (V-ATPase) that acidifies the mineralized tissue, enabling the dissolution of hydroxyapatite crystals (Blair et al., 1989). Other actors, such as cathepsin K, MMP-9, and tartrate-resistant acid phosphatase (TRAP), help degrade bone tissue (Florencio-Silva et al., 2015; Vääräniemi et al., 2004). The products of these degradation reactions are transported by endocytosis into another osteoclast domain.

Blood vessel cells, pericytes, and endothelial cells were closely related to bone physiopathology. As stated earlier, endothelial cells are implicated in tissue remodeling and bone repair, notably through the expression and regulation of proteases that modulate several signaling pathways, such as uPA/uPAR and RAS/MAPK (Heissig et al., 2015). They also contribute to the provision of osteoprogenitor cells (Collin-Osdoby, 1994). Pericytes, also referred to as mural cells, are a population of cells that surround blood vessels. Through direct cell contact, pericytes promote angiogenesis, blood vessel formation, remodeling, and regulation of blood flow (Issabekova et al., 2023). Pericytes expressing CD146+ have been proposed for bone therapy (Harrell et al., 2018). These pericytes can directly differentiate into osteoblasts for bone tissue formation and secrete osteogenic growth factors promoting osteoprogenitor cell proliferation (Nakagomi et al., 2015). *In vivo*, in mice models of periodontitis, pericytes were enriched and more present in periodontal tissues. Pericytes migrate toward the damaged alveolar bone and promote ALP+/OCN+ osteoblasts colonization of the damaged osseous tissue and foster osteogenesis (Cao et al., 2024).

### 2.4 Blood and fibrin in bone formation and regeneration

Blood and bone are closely related from the earliest stages of development. In the embryonic bone, hypertrophic chondrocytes secrete pro-angiogenic factors that promote blood vessel invasion and bone formation (Chen et al., 2020). Perivascular cells and blood-derived cells are mesenchymal, hematopoietic, stem, and progenitor cells that can differentiate into osteogenic and chondrogenic cells and may be the origin of the various cell types found in bone marrow stroma and bone (Chen et al., 2020). Bone is a highly vascularized tissue with an important network of blood vessels, and this vasculature plays a major role in the bone remodeling cycle (Dai and Rabie, 2007). These vessels enable optimal nutrient and oxygen delivery to different locations within the bone, which are required by both HSCs and MSCs for their development. Some long bones can be supplied by many vessels, while smaller bones can be supplied by fewer vessels (Ramasamy, 2017).

**Fig. 4:**
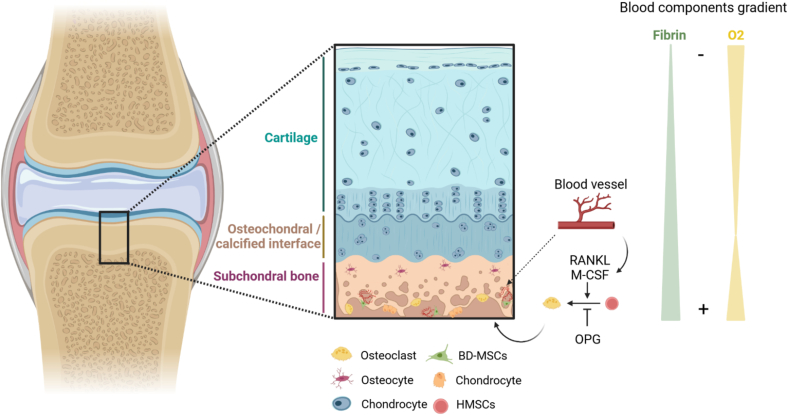
Osteochondral unit showing a fibrin/oxygen gradient from bone to cartilage.

The osteochondral tissue is an “interfacial” tissue between the highly vascularized bone tissue and the “unvascularized” cartilage (Fig. 4). Osteochondral tissue forms a gradient of blood and oxygen supply between bone and cartilage. While cartilage is composed of only one type of cell, the chondrocytes, of mesenchymal origin, bone is composed of cells of mesenchymal (osteoblasts, osteocytes, bone lining cells) and hematopoietic origin (osteoclasts) (Šromová et al., 2023). Hypoxia and hypoxia-inducible factor 1-α (HIF-1α), together with vascularization, play crucial roles in cell differentiation and tissue-specific formation (Šromová et al., 2023). Defects in blood flow in patients with unilateral occlusive disease negatively affect bone mineralization (Laroche et al., 2003). Furthermore, general blood flow defects are considered a potential mechanism for triggering osteoarthritis (Findlay, 2007). Osterix-expressing osteoblast precursors are strictly related to blood vessels during bone production. These precursors showed a perivascular localization, following the direction of the invading blood vessels into the bone (Maes et al., 2010). This indicates that blood vessels play a greater role in bone development than just nutrient supply, making blood a major actor in bone regeneration.

In addition to its structural and physical role in hemostasis, fibrin and its degradation products play various roles in tissue repair, including regulating tissue remodeling and controlling the inflammatory response (Medcalf, 2007; Levi et al., 2004). Fibrin is an essential component of blood, but further studies are needed to understand its biomolecular function in bone physiopathology. In some cases of afibrinogenemia, a condition where patients don't have fibrinogen in their bloodstream, bone pain alongside bone cysts comparable to tumors were observed in the patients (van Meegeren et al., 2014). These cysts usually develop during childhood and are located in the diaphysis of long bones, such as the femur. These patients show “rheumatic” pains in the bone extremities, suggesting a link with osteochondral and cartilage tissues and intraosseous hematoma and hemorrhage (van Meegeren et al., 2014). Cole et al. (2014) proposed that persistent fibrin deposition within the bone matrix could explain inflammation-mediated bone loss (Table 1). Accordingly, they used plasminogen-deficient mice, since plasminogen is responsible for fibrin degradation *in vivo*. Bone metrics, imaging, and histologic evaluation showed that these mice developed osteoporosis with disruption of bone remodeling. These findings established a direct link between bone disease and fibrin, in this mice model (Cole et al., 2014). Furthermore, Raghu and Flick (2011) noted that fibrin could also be a common pathological feature of certain inflammatory disease related to bone, such as rheumatoid arthritis, which makes the distinction between normal hemostasis fibrin and pathological fibrin very critical in studies, as highlighted in Table 1. In rheumatoid arthritis, fibrin deposition actively contributes to the disease progression, acting as a ligand for specific immune cells receptors αMβ2 (Fibγ^390–396A^), driving inflammation (Raghu and Flick, 2011). This statement was confirmed by *Flick* et al. who compared between fibrinogen-deficient arthritis mouse models and normal arthritis mouse models (Flick et al., 2007). The fibrinogen-deficient mice showed fewer affected joints from the arthritis, and reduced disease progression (Flick et al., 2007). To further examine the precise molecular mechanisms, *Flick* et al. included another group of mice expressing a mutant form of fibrinogen only lacking the platelet integrin αIIbβ3-binding motif (Fibγ^Δ5^), while maintaining the clotting function (Flick et al., 2007). The platelet integrin αIIbβ3 mediates calcium responses to shear in platelets (Goncalves et al., 2005). This new group of mice showed no significant difference in disease severity and progression compared to the normal arthritis mice, demonstrating that fibrinogen adhesion to platelets is not an essential driver of arthritis, and that it is strictly context-dependant, depending on its capacity to engage leukocytes through αMβ2 receptor (Flick et al., 2007).

### 2.5 Fibrin-based formulation for osteoarticular repair

**Fig. 5:**
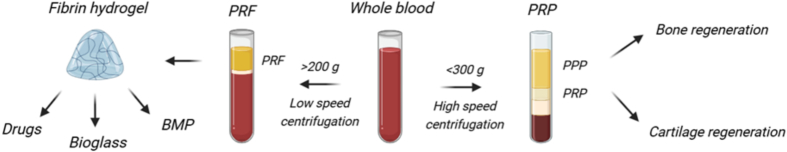
Fibrin-based biomaterial formulations for bone and cartilage tissue repair. The figure illustrates the extraction of PRF from blood to create fibrin hydrogels and PRP for bone and cartilage regeneration.

Fibrin and other fibrin-based formulations, such as platelet-rich fibrin (PRP), have been proposed as therapies for orthopedic and sport-related injuries, including cartilage defects (Kaplonyi et al., 1988; Costa et al., 2025). Fibrin could be used as a hydrogel, as platelet-rich fibrin (PRF), or as platelet-rich plasma (PRP). Both PRP and PRF are forms of plasma, derived from a patient's blood, where PRP is concentrated with low-density fibrin, and PRF is concentrated with high-density fibrin (Fig. 5) (Ao et al., 2020; Jeyachandran et al., 2023; Karfeld-Sulzer et al., 2015; López et al., 2019; Orth et al., 2018; Canbeyli et al., 2018; Xie et al., 2014; Ornetti et al., 2016; Everts et al., 2020). The difference between PRP and PRF lies in the centrifugation speed: PRP is collected at high G force (above 300 g), while PRF is collected at low G force (below 200 g). PRP and PRF are widely used in maxillofacial regeneration, as they are easy to use and rich with pro-angiogenic growth factors (Miron, 2024; Chow et al., 1983; Trevino et al., 2011; Bezgin et al., 2015). A clinical trial involving 113 patients with knee osteoarthritis conducted between 2021 and 2022 showed that microfracture treatment combined with fibrinogen-platelet-rich plasma improved knee joint-related function compared with microfracture alone (Zhang et al., 2024). Moreover, fibrin was studied in the context of tissue engineering. Fibrin promotes bone formation, bone marrow development, and MSC-mediated vascularization, *in vitro* and *in vivo* (Table 2) (Fu et al., 2021; Catelas et al., 2006; Cassaro et al., 2019; Chen et al., 2014; Graziani et al., 2006; Guzel et al., 2015; Ogundipe et al., 2011; Gassling et al., 2013; Li et al., 2013; Du et al., 2018; Yamada et al., 2003; Le Guehennec et al., 2005; Zhao et al., 2022). Some examples reported in Table 2 illustrate that fibrin function in bone repair is not restricted to its formulation as hydrogel, PRP or PRF (Table 2). Further studies are needed to better understand the mechanobiological impact of fibrin formulation for bone repair.

**Table 2:** Fibrin applications in bone regeneration (as fibrin, PRP, PRF, or fibrin composite).

Biomaterial	Model	Levels of evidence	Key limitations
Fibrin	*In vitro* (human MSC) (Catelas et al., 2006) 3D culture of hMSCs seeded in fibrin hydrogels	Cell attachment and proliferation Osteogenic gene expression increase (ALP, RUNX2, COL1A1, assessed by RT-PCR) Osteogenic differentiation by Alizarin Red Staining	Restricted to *in vitro* No osteocalcin expression increase (late osteogenic markers)
	*In vivo* (rat femur) (Cassaro et al., 2019) *n* = 24	New bone formation (*histology*) High osteoconductivity Cell support and ingrowth	No fibrinogen and thrombin concentration variation No osteogenic gene evaluation
	*In vivo* (rabbit femur) (Chen et al., 2014) *n* = 30	High osteogenesis and bone area Cell support Osteopontin expression increased	Limited sample size (10 per condition) No mechanical testing
PRP	*In vitro* (human osteoblasts) (Graziani et al., 2006) 3D culture of human osteoblasts	Cell proliferation Osteogenic marker expression stimulated (*OPN and OC assessed by ELISA*)	Reduced cell viability depending on the PRP concentration Dose-specific effect
	*In vivo* (rat femur) (Guzel et al., 2015) *n* = 40	Histological bone healing High bone strength compared to control	PRP preparation not standardized No dose-response evaluation
	*In vivo* (human molar) (Ogundipe et al., 2011) n = 40	Faster bone healing Reduced swelling post-surgery High bone density	Limited sample size
PRF	*In vitro* (human osteoblasts) (Gassling et al., 2013) 3D culture of human osteoblasts in PRF *vs.* collagen	Cell proliferation High ALP activity (colorimetric assay)	Restricted to *in vitro* No long-term marker evaluated
	*In vitro and in vivo* (human alveolar bone) (Li et al., 2013) n = 24	Cell proliferation and migration Osteogenic gene expression increase (RUNX2, ALP, OPN, OC assessed by RT-PCR) Major bone healing	No fibrinogen and thrombin concentration variation
	*In vivo* (rat alveolar bone) (Du et al., 2018) n = 24	New bone formation Cell proliferation	Small sample size (*8 per condition*)
Fibrin composite	*In vivo* (rat femur) Fibrin + β-TCP (Yamada et al., 2003) n = 30	New bone formation High osteopontin expression (assessed by immunohistochemistry) Critical-size repair	Short observation period No mechanical evaluation No dose-response evaluation
	*In vivo* (rabbit femur) Fibrin + biphasic calcium phosphate (Le Guehennec et al., 2005) *n* = 36	New bone formation Osteoconductivity and osteoinductivity increase	No gene expression evaluation Qualitative data Small sample size (6 per condition)
	*In vivo* (RME rat model) Fibrin + Bioglass (Zhao et al., 2022) *n* = 48	Cell adhesion and proliferation Osteogenic gene expression increase (*RUNX2, ALP assessed by RT-PCR*) Mineralization increase	Only one specific model No mechanical evaluation

It is possible to add additional adjuvant molecules to enhance the effectiveness of the 3D fibrin scaffold for tissue regeneration (Ducret et al., 2021), such as bone morphogenetic proteins 2 and 4 (BMP-2 and BMP-4), which are used for bone tissue engineering, as they promote mineralization and recruit osteoblasts to the damaged bone, especially BMP-2 (Karfeld-Sulzer et al., 2015). BMP-4 specializes in osteogenic induction, promoting the differentiation of osteoprogenitor cells and bone formation (Chen et al., 2021).

In bone tissue engineering, specific biomaterials can be used to regenerate hard osseous tissues, such as calcium phosphate-based materials, including β-TCP (Bohner et al., 2020). Calcium phosphate could also be used in the form of bioglass, as it exhibits high surface activity and produces hydroxyapatite for hard-tissue regeneration (Motta et al., 2023). The hydroxyapatite layer enhances bone regeneration by recruiting osteoblasts and promoting osteoblastic differentiation to form new bone (Motta et al., 2023; Kajave et al., 2021). However, calcium-phosphate materials exhibit low solubility, which limits their penetration into deep sites and reduces their effectiveness at the bone defect site. Encapsulated within a 3D fibrin scaffold, bioglass particles would reach deep tissues, such as alveolar bone, easily, and be delivered directly to the damaged site (Danguir et al., 2026).

In bone repair concepts, interstitial fluid flow through the lacuno-canalicular network is critical for an efficient healing. Osteocytes can sense the mechanical loads caused by the fluid flow, which is a central process in bone homeostasis (Riehl and Lim, 2012). Understanding the link between interstitial fluid flow and the adaptive response of bone is necessary to accordingly develop effective bone healing strategies (Qin and Hu, 2014). If fluid flow is compromised in bone disease states, the mechanical loads are reduced around osteocytes, minimizing the expression of bone anabolic genes and the bone formation (Table 1) (Schurman et al., 2021). At the membrane level, fluid shear stress induces transient PMD in osteocytes, and the rate of these disruptions are repaired and modulates mechanotransduction. With age, or in bone diseases, this PMD is impaired, and consequently, osteocytes lose their ability to sense mechanical loads (Knothe Tate, 2003). The formation of 3D structure by physiological fibrin is regulated *in vivo* by the hydrodynamic shear from physiological flow of blood (Risser et al., 2022). Higher fibrin protein content in blood clot is observed at higher flow (Risser et al., 2022). Intravascular thrombus is also controlled by the physiological shear promoting resistance to fibrinolytic degradation (Whyte and Mutch, 2022). In addition to being a biomolecule whose function is related to blood flow, fibrin is reported to be related to mechanotransduction in platelets, and recently in macrophages in a 3D fibrin biomaterial (Goncalves et al., 2005; Gao et al., 2025). Platelets are able to sense the mechanical properties of their surrounding fibrin matrix through integrin α_IIb_β_3_ and Rac1 signaling (Qiu et al., 2014). This finding demonstrates that fibrin is not merely a structural scaffold, but an active mechanosensory substrate (Qiu et al., 2014). In macrophages, fibrin's specific architecture affects macrophages polarization and deformation through a Tgm2-integrin-pFAK-PGC1α signaling axis (Gao et al., 2025). Macrophage's mitochondria can sense mechanical signals from fibrin through integrins, which activates the Tgm2-pFAK-PGC1α signaling cascade, shifting the macrophage's state, between pro- and anti-inflammatory (Gao et al., 2025). This concept, to date, has never been explored in osteocytes, in bone studies. In contrast, fibrin accumulation in hemarthose suggests a potential toxicity to bone (Charbit et al., 2007). Such as drugs that can directly affect bone composition, it is necessary to assess this aspect in bone repair concepts. Toxic agents can amplify the apoptosis of osteocytes, derailing the bone repair cycle, and glucocorticoids are the most studied example (Table 1) (Gado et al., 2022). These toxic drugs operate through the same fluid flow pathway described earlier, disturbing the whole osteocyte network (Plotkin et al., 1999). These findings establish a fundamental principle: bone toxicity should not be evaluated only by bone density, but also osteocyte viability and properties, with the integrity of the fluid flow kinetics that sustains the whole bone repair concept.

Ultimately, the field of fibrin in bone mechanobiology remains largely unexplored, despite the fact that fibrin is involved in bone physiology and repair. Understanding fibrin's properties in bone mechanobiology is a critical next step to adapt current treatments, and improve them.

## 3 Conclusion

All of these data suggest that fibrin sustains many vital functions in the human body such as hemostasis, coagulation, and wound healing. Beyond its classical role in the formation of a blood clot, fibrin also provides a temporary extracellular matrix that orchestrates the early stages of tissue repair. The pathophysiological function of fibrin is related to blood flow and mechanobiology on platelets. This review highlights the critical gap of knowledge between fibrin biomolecule in blood and bone mechanobiology. Consequently, this biopolymer is a multifunctional natural biomolecule, at the crossroads of many biological mechanisms including inflammation and tissue remodeling that might be further investigated in mechanobiology to finely tune future therapeutic strategies for bone diagnostic and repair. Even though fibrin is currently already used in bone regeneration, a deeper investigation of fibrin's role in bone mechanobiology, in both healthy and pathological cases, is necessary, to ultimately improve both the diagnosis and treatment of bone diseases in which fluid flow and mechanosensing converge.

## CRediT authorship contribution statement

**Hamza Danguir:** Writing – original draft, Formal analysis, Data curation. **Lisa Reiniche:** Writing – review & editing, Visualization. **Khalil El Mabrouk:** Writing – review & editing, Resources. **Maxime Ducret:** Writing – review & editing, Formal analysis. **Meriame Bricha:** Writing – review & editing, Supervision, Data curation. **Mourad Bekhouche:** Writing – review & editing, Visualization, Supervision, Funding acquisition.

## Funding

The authors gratefully acknowledge 10.13039/501100006537Campus France for their financial support with a “Partenariat Hubert Curien” (PHC) grant (PHC TOUBKAL, TBK/24/198, 49978NB). We gratefully acknowledge Lyon 1 University (UCBL, AAP Accueil EC 2024-2025), the Laboratory of Tissue Biology and therapeutic Engineering (LBTI, UMR CNRS 5305) and Euromed University of Fes (UEMF) for their financial and administrative assistance in making this research project possible. We thank the French Institute for ondontological Research (IFRO) for their financial support. This work was funded by the French National Centre for Scientific Research (CNRS), and the 10.13039/501100001665French National Research Agency (Tridentomic projet, N°ANR-21-CE44-0015-01, Endonanobiotic project, N°ANR-21-CE19-0001, and PULPITOMIC, N°ANR-25-CE44-4117-01).

## Declaration of competing interest

The authors declare no conflict of interest.

## Data Availability

No data was used for the research described in the article.
